# Short- and mid-term outcomes of mitral valve repair vs. replacement in degenerative mitral regurgitation with left ventricular enlargement

**DOI:** 10.3389/fcvm.2026.1944441

**Published:** 2026-09-25

**Authors:** Chun Wu, Zhe Sheng, Jianhao Hu, Yuyong Liu, Chengxin Zhang, Jinguo Xu

**Affiliations:** 1 Department of Cardiovascular Surgery, The First Affiliated Hospital of Anhui Medical University, Hefei, China; 2 Department of Cardiac Ultrasound Imaging Center, The First Affiliated Hospital of Anhui Medical University, Hefei, China; 3 Beijing Institute of Heart, Lung and Blood Vessel Diseases, Beijing Anzhen Hospital Affiliated to Capital Medical University, Beijing, China

**Keywords:** dilated left ventricle, global longitudinal strain, mitral valve replacement, mitral valve regurgitation, mitral valve repair

## Abstract

**Objective:**

This study evaluates short- to mid-term outcomes following mitral valve repair (MVP) and mitral valve replacement (MVR) among patients with degenerative mitral regurgitation (DMR) complicated by substantial left ventricular dilatation (LVD).

**Methods:**

We performed a retrospective analysis enrolling 150 patients diagnosed with DMR and severe LVD who received either MVP or MVR. The median clinical follow-up duration was 55.5 (21.0) months. Propensity score matching (PSM) was conducted to eliminate intergroup baseline disparities. Kaplan–Meier survival curves were generated to estimate short- and mid-term all-cause survival.

**Results:**

Of the 150 enrolled patients with DMR and marked left ventricular dilatation, 56.67% underwent MVR and 43.33% received MVP. The cohort had a median age of 59 (17) years, with female patients making up 23.3% of participants. Preoperative atrial fibrillation occurred in 15.33% of patients, and 92.05% presented with severe baseline mitral valve regurgitation (MR). Analyses before and after PSM showed similar postoperative left ventricular end-diastolic dimension (LVEDD) and New York Heart Association functional class across groups. Significant intergroup differences were detected in postoperative MR (*P* = 0.017), left ventricular ejection fraction (LVEF) (*P* = 0.005), and global longitudinal strain (GLS) (*P* = 0.023). Kaplan–Meier curves indicated equivalent postoperative survival for patients who underwent MVP and MVR both before matching (log-rank *P* = 0.175) and after PSM matching (log-rank *P* = 0.497).

**Conclusions:**

In patients with DMR and dilated left ventricles (≥6.0 cm), MVR was associated with a greater incidence of postoperative hemorrhage related to long-term oral anticoagulation, whereas MVP conferred a higher risk of recurrent MR. Postoperative mortality was comparable across cohorts; however, preserved left ventricular subclinical systolic function (GLS and LVEF) in the MVP group suggests potential long-term myocardial benefits, which warrants further prospective investigation.

## Introduction

In degenerative mitral regurgitation (DMR), structural degeneration of the mitral apparatus compromises leaflet coaptation, resulting in systolic regurgitation. This imposes a chronic volume overload on the left heart, precipitating remodeling that manifests as severe left ventricular dilation (LVD) or left atrial dilation, pulmonary hypertension, and heart failure (1). Driven by population growth and unhealthy living habits, the disease burden has risen substantially (2). Over the past two decades, its documented prevalence has increased by approximately 70%, reaching an estimated 18.1 million cases by 2017 (3, 4). Guidelines mandate surgery for symptomatic severe DMR, favoring repair over replacement due to its association with better preservation of left ventricular function and survival (5, 6). Mitral valve repair (MVP) preserves native tissue, maintains left ventricular function, and supports favorable reverse remodeling. While often performed minimally invasively, the major advantage of MVP is the avoidance of lifelong anticoagulation, significantly reducing bleeding and thromboembolic risks for the patient. Overall, MVP is associated with superior survival, lower operative mortality, and fewer valve-related complications than mitral valve replacement (MVR) (7–9). While early operative mortality after MVR has been documented as an independent risk factor in high-risk elderly populations (10), its relative performance compared with MVP in younger cohorts presenting with advanced LVD remains to be systematically defined. Moreover, in this setting, replacement typically requires lifelong oral anticoagulation, introducing a competing risk of major bleeding, whereas repair carries a higher risk of recurrent mitral regurgitation; the optimal trade-off between these two hazards remains unresolved.

The anatomical defects from complex remodeling in DMR with marked LVD are significant predictors of postoperative mitral valve regurgitation (MR) recurrence (11). To address this clinical uncertainty, we retrospectively analyzed patients at our institution who underwent surgery for DMR with advanced left ventricular dilation.

## Materials and methods

### Study design

We retrospectively analyzed 150 consecutive patients with DMR and a left ventricular end-diastolic dimension (LVEDD) ≥6.0 cm who underwent MVP or MVR (The First Affiliated Hospital of Anhui Medical University, October 2017–January 2023). MR and tricuspid regurgitation severity were graded according to standard guidelines (12). This study enrolled patients who underwent primary surgery for DMR. Recurrent MR was defined as a postoperative severity of moderate or greater. Exclusion criteria comprised non-degenerative etiologies, concomitant aortic valve procedures, reoperations, or coronary artery bypass grafting. The primary endpoint was all-cause mortality, with secondary endpoints including perioperative adverse events, valve-related reoperation, and major complications such as hemorrhage.

### Patient cohort

Patients were stratified into the MVP or MVR group based on their surgical procedure. To minimize confounding, propensity score matching (PSM) was applied, and outcomes were analyzed both before and after matching.

### Perioperative assessment and surgical strategy

All patients received guideline-directed medical therapy before surgery. The diagnosis of DMR was established based on clinical evaluation and transthoracic echocardiography (TTE). An initial surgical plan was formulated preoperatively, and informed consent was obtained for either MVP or MVR. The final surgical strategy was determined intraoperatively by an experienced multidisciplinary heart team based on direct assessment of leaflet pathology, anatomical feasibility, and the extent of left ventricular remodeling. MVP was the preferred strategy whenever a durable repair was deemed technically achievable, particularly in patients with isolated posterior leaflet prolapse or localized leaflet defects. MVR was favored when a durable repair was unlikely, including cases with extensive leaflet destruction, severe calcification, complex bileaflet or anterior leaflet prolapse not amenable to repair, or severe leaflet tethering associated with advanced ventricular remodeling. Thus, the choice between MVP and MVR was primarily guided by the anticipated durability of the repair rather than anatomical severity alone.

### Surgical technique

Surgery was performed through either standard median sternotomy or minimally invasive approaches, including right mini-thoracotomy, total thoracoscopic surgery, and robot-assisted surgery, according to institutional practice and patient-specific considerations. In the MVP cohort, the repair strategy was tailored to the underlying leaflet pathology and included triangular or rectangular leaflet resection, artificial chordae tendineae implantation, commissural closure, and prosthetic annuloplasty. Annuloplasty was performed using rigid or semi-rigid complete rings or C-shaped rings. In patients with severe ventricular dilation, the ring was routinely undersized by 1–2 sizes relative to the anterior leaflet area to optimize leaflet coaptation and enhance repair durability. In the MVR cohort, prosthetic valve replacement was performed with preservation of the subvalvular apparatus whenever feasible, including complete preservation of the posterior leaflet and associated chordae tendineae. Before PSM, the median annuloplasty ring size in the MVP group was 30 (2) mm, and the median prosthetic valve size in the MVR group was 27 (1) mm. Following PSM, the corresponding median ring and valve sizes were 30 (3) mm and 27 (2) mm, respectively (Figure 1). Mechanical prostheses were implanted in 56 patients (65.88%), whereas bioprosthetic valves were used in 29 patients (34.12%), based on patient age, comorbidities, and anticoagulation considerations.

**Figure 1:**
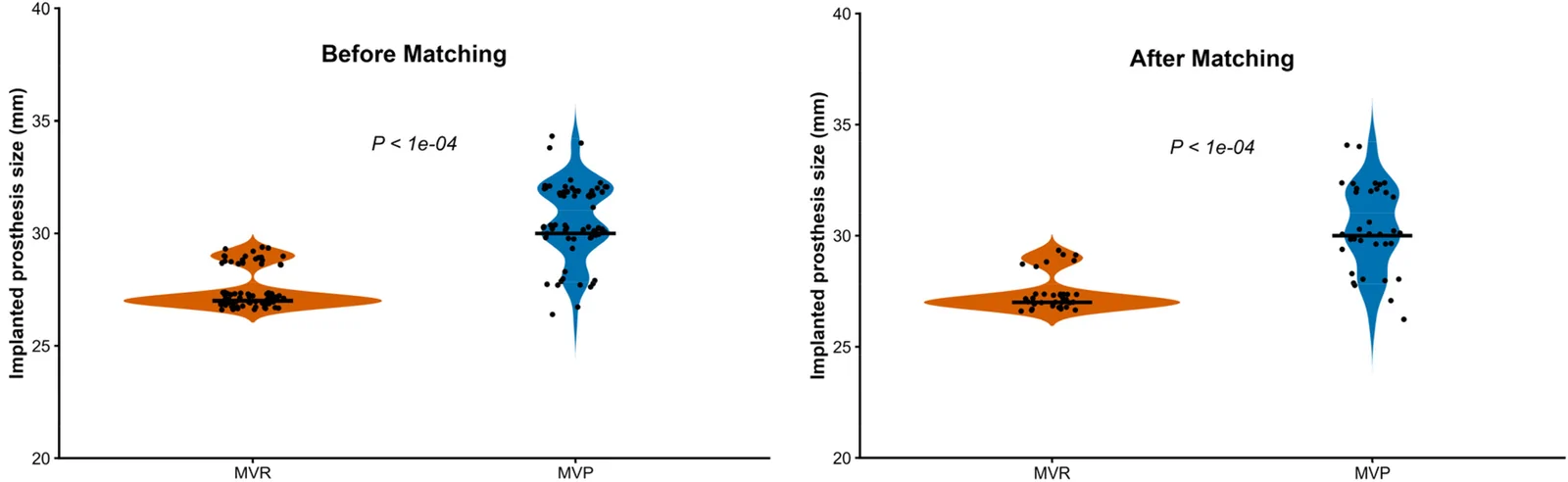
Comparison of implanted prosthetic valve size between the MVR and MVP groups. Data analysis was performed using the non-parametric Mann–Whitney *U* test.

### Intraoperative assessment and concomitant procedures

Intraoperative transesophageal echocardiography was routinely performed to assess the adequacy of the mitral valve repair or prosthetic valve function before weaning from cardiopulmonary bypass. Reintervention was performed when residual moderate or greater mitral regurgitation was identified. Concomitant tricuspid valve repair was performed when indicated, using either the De Vega technique or prosthetic annuloplasty rings. All procedures were performed by experienced surgeons.

### Postoperative anticoagulation protocol and bleeding definition

All patients in the MVR group who received mechanical valves received lifelong oral anticoagulation with warfarin, targeting an international normalized ratio (INR) of 2.0–3.0. Patients who received bioprosthetic valves or underwent MVP received oral warfarin for 3 months postoperatively (target INR 1.5–2.5), followed by long-term antiplatelet therapy or no antithrombotic therapy if in sinus rhythm. Postoperative hemorrhagic events were defined according to the Bleeding Academic Research Consortium (BARC) criteria (grade ≥2 bleeding), including overt clinically significant bleeding requiring hospitalization, blood transfusion, or invasive intervention (e.g., severe hematochezia, epistaxis requiring packing/cautery, or gastrointestinal bleeding).

### Serial echocardiographic evaluation and global longitudinal strain protocol

Serial TTE assessments were performed preoperatively, at hospital discharge, and during scheduled post-discharge outpatient follow-up. The follow-up TTE parameters reported in Table 1 were obtained from the most recent available outpatient echocardiogram for each patient at the time of data extraction, rather than at a single protocol-defined fixed time point. Standard cardiac dimensions, left ventricular ejection fraction (LVEF; via Simpson's biplane method), and regurgitation severity were assessed. Global longitudinal strain (GLS) was measured offline using automated speckle-tracking echocardiography from apical 4-chamber, 2-chamber, and long-axis views.

**Table 1:** Follow-up TTE parameters and adverse events.

	Before PSM				After PSM			
Variable	Total (*N* = 141)	MVR (*N* = 77)	MVP (*N* = 64)	*P*-value	Total (*N* = 67)	MVR (*N* = 32)	MVP (*N* = 35)	*P*-value
Valve-related reoperation, *N*	2 (1.42%)	0 (0%)	2 (3.13%)	0.204	2 (2.99%)	0 (0%)	2 (5.7%)	0.493
Bleeding events	8 (5.67%)	8 (10.39%)	0 (0%)	0.020	6 (8.96%)	6 (18.75%)	0 (0%)	0.024
LAD (cm)	4.34 (0.44)	4.35 (0.44)	4.31 (0.43)	0.988	4.36 (0.47)	4.37 (0.46)	4.36 (0.52)	0.821
LVEDD (cm)	5.35 (0.50)	5.37 (0.45)	5.34 (0.54)	0.998	5.34 (0.52)	5.39 (0.42)	5.32 (0.58)	0.826
LVEF (%)	59 (7)	58 (7)	59.5 (7)	0.006	59 (7)	57 (9)	60 (7)	0.005
LVSV (mL)	82.91 ± 13.01	83.95 ± 12.96	81.66 ± 13.08	0.300	84.18 ± 13.59	84.19 ± 13.85	84.17 ± 13.55	0.996
LVFS (%)	32 (4)	31 (4)	32 (3)	0.038	32 (4)	30.5 (4)	32 (3)	0.027
GLS (%)	−18.1 (2.6)	−17.4 (2.3)	−18.75 (2.6)	<0.001	−17.96 ± 1.83	−17.43 (1.67)	−18.44 (1.88)	0.023
NYHA class				0.090				0.096
Ⅰ	102 (72.34%)	52 (67.53%)	50 (78.12%)		49 (73.13%)	21 (65.63%)	28 (80%)	
Ⅱ	22 (15.60%)	11 (14.29%)	11 (17.19%)		13 (19.40%)	6 (18.75%)	7 (20%)	
Ⅲ	11 (7.80%)	9 (11.69%)	2 (3.13%)		2 (2.99%)	2 (6.25%)	0 (0%)	
Ⅳ	6 (4.26%)	5 (6.49%)	1 (1.56%)		3 (4.48%)	3 (9.38%)	0 (0%)	
MR severity				0.002				0.017
Mild or less	120 (85.11%)	72 (93.51%)	48 (75%)		55 (82.09%)	30 (93.75%)	25 (71.43%)	
Moderate	19 (13.47%)	5 (6.49%)	14 (21.88%)		11 (16.42%)	2 (6.25%)	9 (25.71%)	
Severe	2 (1.42%)	0 (0%)	2 (3.12%)		1 (1.49%)	0 (0%)	1 (2.86%)	
TR severity				0.723				0.335
Mild or less	131 (92.91%)	71 (92.21%)	60 (93.75%)		61 (91.04%)	28 (87.5%)	33 (94.29%)	
Moderate	10 (7.09%)	6 (7.79%)	4 (6.25%)		6 (8.96%)	4 (12.5%)	2 (5.71%)	

LAD, left atrial dimension; LVEDD, left ventricular end-diastolic dimension; LVEF, left ventricular ejection fraction; LVSV, left ventricular stroke volume; LVFS, left ventricular fractional shortening; GLS, global longitudinal strain; NYHA, New York Heart Association; MR, mitral regurgitation; TR, tricuspid regurgitation. Categorical variables are presented as number (percentage), *n* (%). For continuous variables, non-normally distributed data are described as median (interquartile range, IQR), and normally distributed data are described as mean ± standard deviation (SD).

### Statistical analysis

Statistical analyses were performed using SPSS 26.0, R 4.4.2, and RStudio (2024.12.1 Build 563). The normality of the continuous data was evaluated using the Shapiro–Wilk test. Continuous variables conforming to a normal distribution are expressed as mean ± SD and were compared using Student's t-test; non-normally distributed continuous variables are presented as median (IQR) and were evaluated using the Mann–Whitney *U* test. Categorical data [n (%)] were compared with the *χ*^2^ or Fisher's exact test. PSM (1:1 nearest-neighbor, caliper = 0.02) incorporated gender; age; body mass index (BMI); hypertension; New York Heart Association class; atrial fibrillation; and preoperative echocardiographic parameters including left atrial dimension, LVEDD, LVEF, left ventricular stroke volume, left ventricular fractional shortening, MR, and tricuspid regurgitation. Survival was analyzed using Kaplan–Meier curves and the log-rank test. A two-sided *P-*value < 0.05 indicated statistical significance. Because clinical follow-up differed between the surgical groups, we performed two sensitivity analyses to verify that the null survival finding was not an artifact of differential censoring or potential non-proportional hazards, as follows: (i) a fixed-window (landmark) log-rank test with both groups censored at 48 and 60 months, and (ii) restricted mean survival time (RMST) at *τ* = 60 months, reported as the MVR−MVP difference with its 95% confidence interval.

## Results

### Baseline characteristics

This study included 150 patients (MVR: 85, 56.7%; MVP: 65, 43.3%) with a median age of 59 (17) years; 23.3% were female. All patients had advanced LVD and 92.05% had severe MR. Before PSM, significant differences existed in gender (*P* = 0.005), BMI (*P* = 0.001), left atrial dimension (*P* = 0.004), LVEF (*P* = 0.016), and tricuspid regurgitation (*P* = 0.039). Thereafter, PSM successfully balanced all baseline characteristics, as shown by standardized mean differences and the distribution of propensity scores (Table 2, Figures 2, 3).

**Table 2:** Baseline patient characteristics.

	Before PSM				After PSM			
Variable	Total (*N* = 150)	MVR (*N* = 85)	MVP (*N* = 65)	*P*-value	Total (*N* = 72)	MVR (*N* = 36)	MVP (*N* = 36)	*P*-value
Gender (female)	35 (23.3%)	27 (31.76%)	8 (12.31%)	0.005	15 (20.8%)	6 (16.7%)	9 (25.0%)	0.384
Age (years)	59 (17)	58 (16)	59 (16)	0.841	58.24 (11.38)	57.86 (10.10)	58.61 (12.66)	0.782
Hypertension	19 (12.67%)	12 (14.12%)	7 (10.77%)	0.541	10 (13.9%)	5 (13.9%)	5 (13.9%	1.000
Diabetes mellitus	14 (9.33%)	9 (10.59%)	5 (7.69%)	0.546	11 (15.28%)	6 (16.67%)	5 (13.89%)	0.743
BMI	23.50 ± 4.25	22.53 ± 3.95	24.77 ± 4.32	0.001	23.56 ± 3.53	23.70 ± 3.91	23.41 ± 3.15	0.733
NYHA class				0.100				1.000
Ⅲ	128 (85.33%)	69 (81.18%)	59 (90.77%)		64 (88.89%)	32 (88.9%)	32 (88.9%)	
Ⅳ	22 (14.67%)	16 (18.82%)	6 (9.23%)		8 (11.11%)	4 (11.1%)	4 (11.1%)	
AF	23 (15.33%)	16 (18.82%)	7 (10.77%)	0.175	8 (11.11%)	3 (8.33%)	5 (13.89%)	0.708
LAD (cm)	5.54 (1.14)	5.83 (1.43)	5.3 (1.09)	0.004	5.59 (1.00)	5.60 (1.17)	5.57 (0.96)	0.783
LVEDD (cm)	6.41 (0.71)	6.44 (0.73)	6.36 (0.64)	0.091	6.44 (0.71)	6.39 (0.75)	6.54 (0.71)	0.778
LVEF (%)	60 (8)	58 (8)	60 (7)	0.016	60 (7)	61 (7)	60 (9)	0.635
LVSV (mL)	121 (37)	121 (42)	120 (30)	0.749	126.50 (34)	123 (42)	127.50 (32)	0.813
LVFS (%)	32 (4)	32 (4)	33 (5)	0.379	33 (5)	33 (5)	32 (4)	0.325
TR treatment	139 (92.67%)	82 (96.47%)	57 (87.69%)	0.084	69 (95.83%)	36 (100%)	33 (91.67%)	0.238
MR severity				0.866				0.607
Moderate	11 (7.33%)	7 (8.14%)	4 (6.15%)		4 (5.56%)	1 (2.78%)	3 (8.33%)	
Severe	139 (92.05%)	78 (90.70%)	61 (93.85%)		68 (94.44%)	35 (97.2%)	33 (91.7%)	
TR severity				0.039				0.759
Mild or less	102 (68%)	52 (61.18%)	50 (76.92%)		53 (73.61%)	26 (72.22%)	27 (75%)	
Moderate	37 (24.67%)	25 (29.41%)	12 (18.46%)		12 (16.67%)	6 (16.67%)	6 (16.67%)	
Severe	11 (7.33%)	8 (9.41%)	3 (4.62%)		7 (9.72%)	4 (11.11%)	3 (8.33%)	

BMI, body mass index; NYHA, New York Heart Association; AF, atrial fibrillation; LAD, left atrial dimension; LVEDD, left ventricular end-diastolic dimension; LVEF, left ventricular ejection fraction; LVSV, left ventricular stroke volume; LVFS, left ventricular fractional shortening; MR, mitral regurgitation; TR, tricuspid regurgitation. Categorical variables are presented as number (percentage), *n* (%). For continuous variables, non-normally distributed data are described as median (interquartile range, IQR), and normally distributed data are described as mean ± standard deviation (SD).

**Figure 2:**
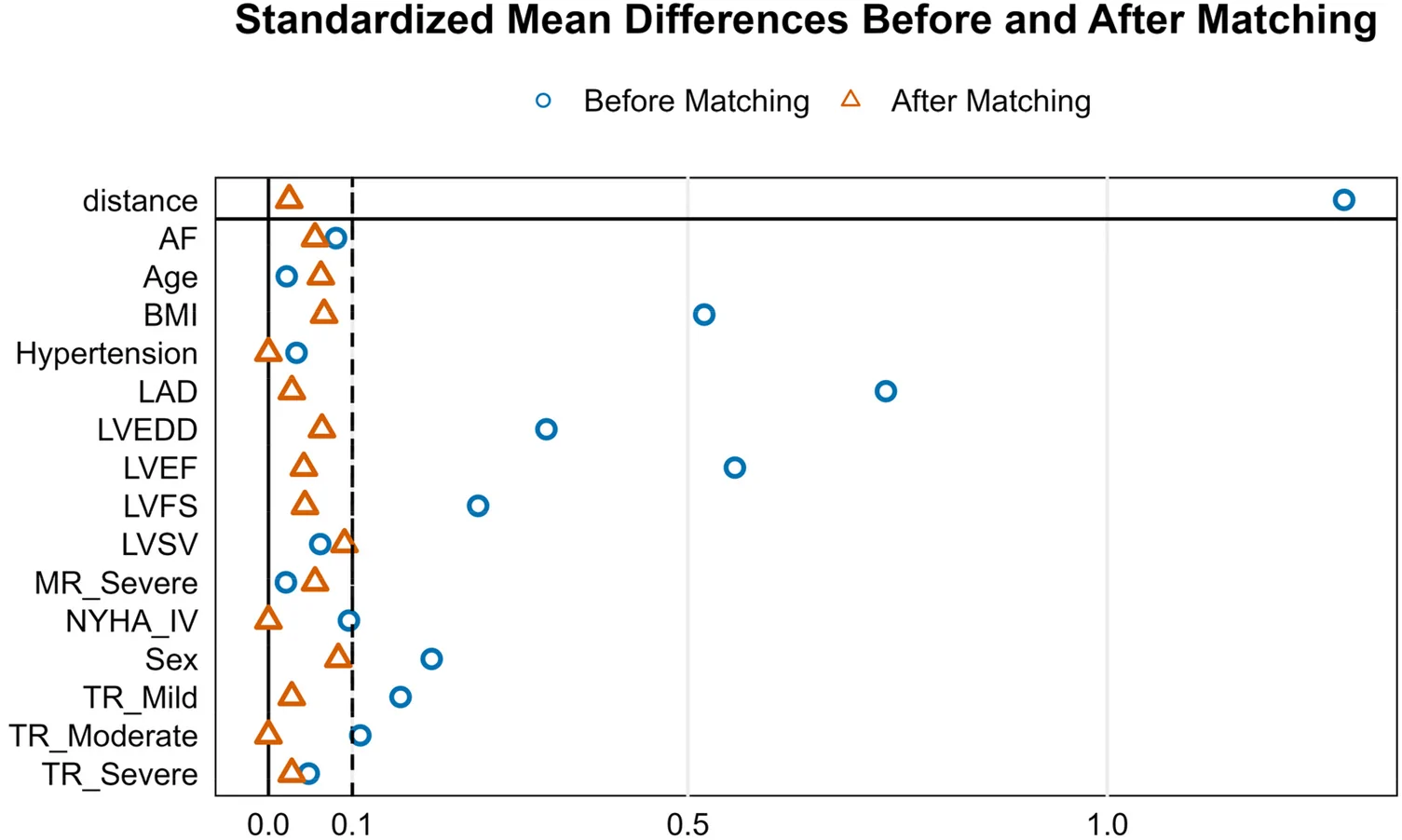
Standardized mean differences (SMDs) comparing baseline characteristics between the MVP and MVR groups. An SMD below 10% indicates a balanced distribution of covariates between the groups. AF, atrial fibrillation; BMI, body mass index; LAD, left atrial dimension; LVEDD, left ventricular end-diastolic dimension; LVEF, left ventricular ejection fraction; LVFS, left ventricular fractional shortening; LVSV, left ventricular stroke volume; MR, mitral regurgitation; NYHA, New York Heart Association; TR, tricuspid regurgitation.

**Figure 3:**
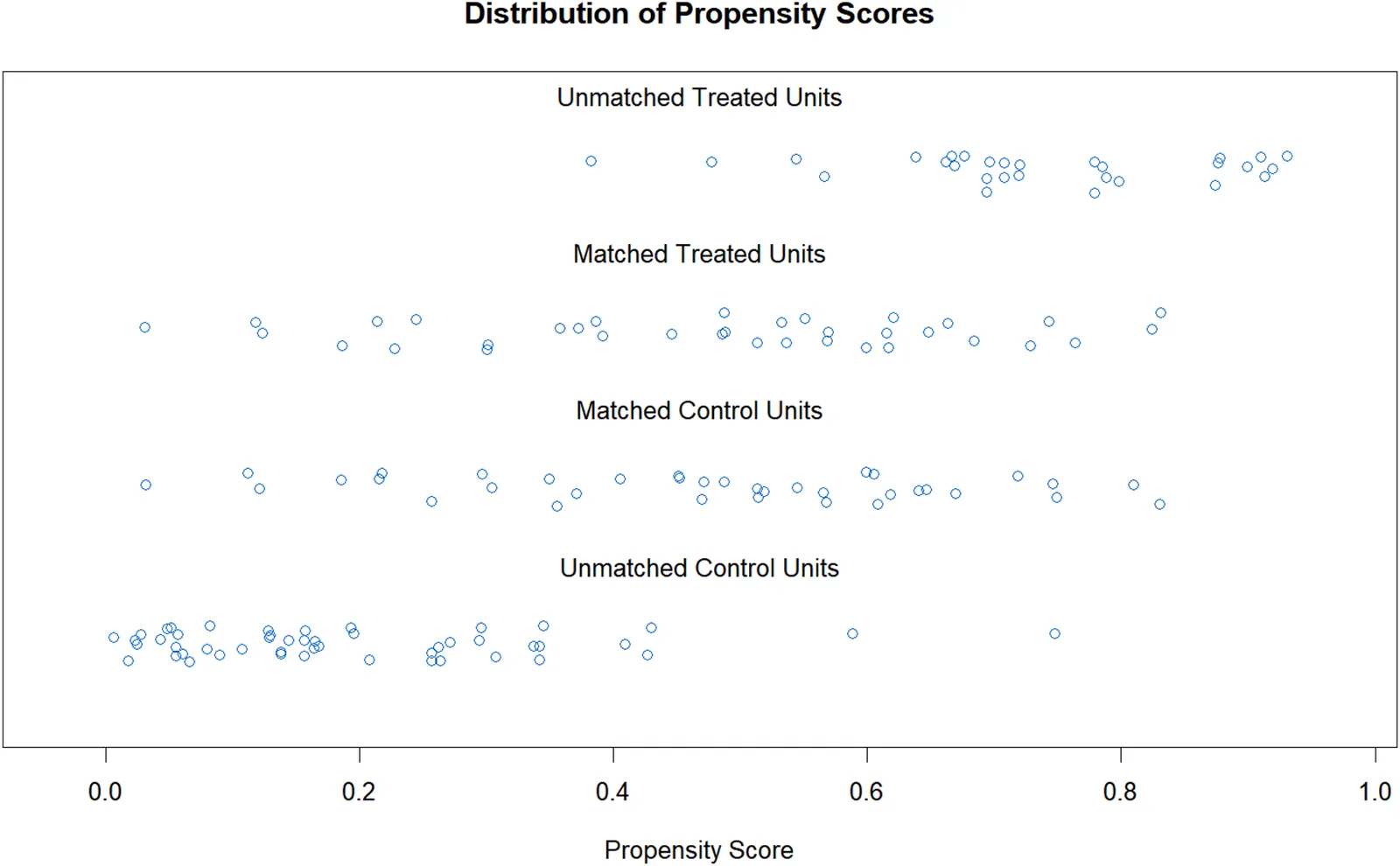
Distribution of propensity scores before and after matching.

### Perioperative characteristics and discharged TTE parameters

Pre-PSM, significant differences were found in discharge left atrial dimension (*P* = 0.008) and LVEF (*P* = 0.020). Post-PSM, echocardiographic parameters at discharge demonstrated no significant differences (Table 3).

**Table 3:** Perioperative characteristics and discharged TTE parameters.

	Before PSM				After PSM			
Variable	Total (*N* = 150)	MVR (*N* = 85)	MVP (*N* = 65)	*P*-value	Total (*N* = 72)	MVR (*N* = 36)	MVP (*N* = 36)	*P*-value
Hospital stay (days)	15 (6)	15 (5)	14 (5)	0.003	14.5 (5)	15 (5)	14 (5)	0.020
Ventilation time (h)	18.5 (4)	19 (4)	18 (3)	0.004	18.86 ± 2.84	19.69 ± 2.85	18.03 ± 2.61	0.012
ICU stay (h)	24 (4)	25 (3.5)	23 (4)	<0.001	25 (4)	25 (3)	24 (5)	0.045
CPB time (min)	121.67 ± 12.56	121.48 ± 13.85	121.89 ± 10.74	0.844	120.69 ± 13.61	120.67 ± 15.38	120.72 ± 11.8	0.986
ACC time (min)	86.79 ± 9.80	88.27 ± 9.16	84.86 ± 10.33	0.034	85.78 ± 10.02	88.67 ± 9.11	84.89 ± 10.91	0.456
LAD (cm)	4.61 (1.05)	4.74 (1.21)	4.41 (0.75)	0.008	4.66 (0.99)	4.71 (1.21)	4.58 (0.75)	0.532
LVEDD (cm)	5.57 ± 0.51	5.63 ± 0.51	5.49 ± 0.50	0.100	5.57 ± 0.51	5.56 ± 0.51	5.58 ± 0.50	0.839
LVEF (%)	57.5 (7)	56 (6)	58 (8)	0.020	58 (5)	58 (5)	58 (8)	0.781
LVSV (mL)	84 (22)	84 (23)	83 (23)	0.316	85.50 (24)	85.50 (23)	85.50 (28)	0.573
LVFS (%)	31 (3)	30 (3)	31 (5)	0.170	31 (2)	31 (2)	31 (5)	0.320
MR severity				1.000				1.000
Mild or less	150 (100%)	85 (100%)	65 (100%)		72 (100%)	36 (100%)	36 (100%)	
Moderate	0 (0)	0 (0)	0 (0)		0 (0)	0 (0)	0 (0)	
Severe	0 (0)	0 (0)	0 (0)		0 (0)	0 (0)	0 (0)	
TR severity				1.000				1.000
Mild or less	150 (100%)	85 (100%)	65 (100%)		72 (100%)	36 (100%)	36 (100%)	
Moderate	0 (0)	0 (0)	0 (0)		0 (0)	0 (0)	0 (0)	
Severe	0 (0)	0 (0)	0 (0)		0 (0)	0 (0)	0 (0)	

ICU, intensive care unit; CPB, cardiopulmonary bypass; ACC, aortic cross-clamp; LAD, left atrial dimension; LVEDD, left ventricular end-diastolic dimension; LVEF, left ventricular ejection fraction; LVSV, left ventricular stroke volume; LVFS, left ventricular fractional shortening; MR, mitral regurgitation; TR, tricuspid regurgitation. Categorical variables are presented as number (percentage), *n* (%). For continuous variables, non-normally distributed data are described as median (interquartile range, IQR), and normally distributed data are described as mean ± standard deviation (SD).

### All-cause mortality and follow-up durations

Kaplan–Meier analysis revealed no significant difference in survival between the MVR and MVP groups either before or after PSM (Figure 4). Although the MVR group had more deaths than the MVP group (8 vs. 1 before matching; 4 vs. 1 after matching), these deaths were mainly from hemorrhagic and thromboembolic causes, with no perioperative mortality. This higher number of deaths was accompanied by longer clinical follow-up in the MVR group, both before matching [59.0 (24.0) vs. 52.0 (22.0) months; *P* < 0.001] and after matching [55.0 (20.2) vs. 48.5 (25.5) months; *P* = 0.019]. As the patients who underwent MVR contributed more person-time at risk, the observed survival equivalence is conservative for MVR and not a consequence of informative censoring.

**Figure 4:**
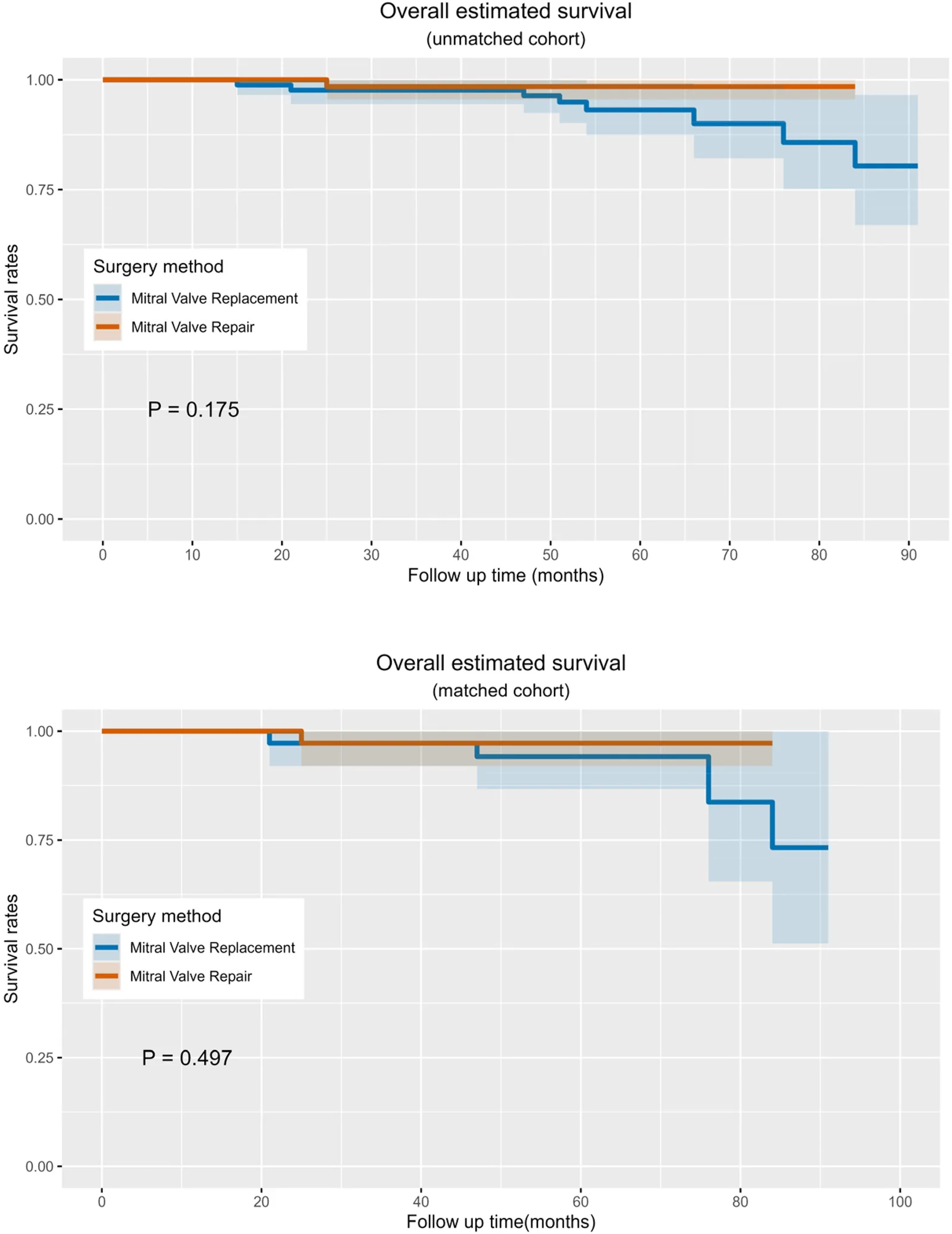
Before and after PSM Kaplan–Meier analysis curves.

Sensitivity analyses further supported this interpretation. Fixed-window landmark log-rank tests censoring follow-up at 48 and 60 months remained non-significant (before matching, *P* = 0.504 and *P* = 0.260, respectively; after matching, *P* = 0.658 at both windows). Similarly, the between-group difference in 60-month restricted mean survival time was negligible [before matching, −0.85 months (95% CI, −2.62 to 0.91), *P* = 0.34; after matching, −0.51 months (95% CI, −3.41 to 2.39), *P* = 0.73] (Table 4).

**Table 4:** Clinical follow-up duration by surgical group and sensitivity analyses for post-surgical survival.

a. Clinical follow-up by surgical group (before and after propensity score matching)			
Cohort	Group	*N*	Clinical follow-up, median (IQR), months
Before PSM	MVR	85	59.0 (24.0)
Before PSM	MVP	65	52.0 (22.0)
After PSM	MVR	36	55.0 (20.2)
After PSM	MVP	36	48.5 (25.5)

b. Sensitivity analyses for post-surgical survival (fixed-window landmark log-rank and RMST)					
Cohort	Landmark window (months)	Landmark log-rank p	RMST difference (MVR MVP), months	RMST 95% CI	RMST *P*-value
Before PSM	48	0.504	−0.37	−1.56–0.83	0.55
Before PSM	60	0.260	−0.85	−2.62–0.91	0.34
After PSM	48	0.658	−0.14	−2.05–1.76	0.88
After PSM	60	0.658	−0.51	−3.41–2.39	0.73

Clinical follow-up was defined as the interval from the date of surgery to all-cause death or last documented clinical contact, and is the time scale of the Kaplan–Meier survival analysis. Values are median (interquartile range, IQR). The between-group difference in clinical follow-up (MVR vs. MVP), calculated using the Mann–Whitney *U* test, was *P* < 0.001 before PSM and *P* = 0.019 after PSM. The landmark analysis censored both groups at the specified window. RMST, restricted mean survival time to tau; RMST difference is MVR–MVP. All 95% confidence intervals cross zero and all *P* > 0.3, confirming the null survival result is not driven by the between-group follow-up imbalance.

### Hemorrhage and reoperation

No hemorrhagic events occurred in the MVP group. In contrast, major hemorrhagic events (BARC grade ≥2) were recorded in the MVR group [eight events (10.39%) pre-PSM and six events (18.75%) post-PSM; *P* = 0.020 and *P* = 0.024, respectively], manifesting as gastrointestinal bleeding/hematochezia or severe epistaxis, closely associated with long-term warfarin anticoagulation therapy.

Regarding reoperations, two patients who underwent MVP required surgery for recurrent severe regurgitation, whereas none were required in the MVR group (pre-PSM: *P* = 0.204; post-PSM: *P* = 0.493).

### Follow-up TTE parameters

Postoperative TTE demonstrated that the MVP group had persistently higher MR severity and significant differences in LVEF and GLS. However, this did not translate to a difference in NYHA functional class. During follow-up, complete echocardiographic data were not obtainable for nine patients (eight in the pre-PSM MVR group and one in the MVP group; four in the post-matching MVR group and one in the MVP group) due to death or loss to follow-up. Accordingly, the sample size for TTE analysis was reduced to 141 cases (unmatched cohort) and 67 cases (post-matching cohort) (Table 1). The follow-up TTE parameters in Table 1 represent the most recent available outpatient echocardiogram for each patient at the time of data extraction.

## Discussion

In this study, we compared outcomes following MVP vs. MVR. The key findings are as follows: (1) no significant difference in postoperative all-cause mortality was observed between groups, before or after PSM; (2) valve-related reoperation rates were similar, but the patients who underwent MVP had a significantly higher recurrence of MR, whereas those who underwent MVR experienced more hemorrhagic complications; and (3) significant differences were noted in LV systolic function—LVEF and GLS—between groups.

Traditionally, MVP has been the preferred surgical approach for DMR, as it preserves the native valve apparatus and is associated with superior long-term survival and fewer valve-related complications compared to MVR (13, 14). However, its efficacy in patients with pre-existing advanced LVD remains uncertain, as postoperative ventricular remodeling may alter the anatomical and functional suitability of the repair. Moreover, DMR with significant left ventricular enlargement is itself an independent risk factor for reduced long-term survival, raising questions about the durability and survival benefit of MVP in this population (15).

Previous studies have suggested a survival advantage for MVP in unadjusted cohorts, but this benefit was attenuated after adjusting for baseline differences using PSM (16). While this is a study of ischemic MR, our cohort-specific analysis of DMR with advanced LVD also showed no significant difference in postoperative survival between MVP and MVR, both pre-PSM (log-rank *P* = 0.175) and post-PSM (log-rank *P* = 0.497).

MVP aims to preserve the native valve tissue, while MVR replaces the pathological valve with an artificial prosthesis. As DMR is a progressive condition driven by myxomatous or fibroelastotic degeneration, the native valve may continue to degenerate even after a technically successful repair. This ongoing pathology, such as leaflet thickening, elongation, or new chordal rupture, increases the risk of recurrent regurgitation. Therefore, MVP carries a higher risk of recurrence compared to MVR (17). Anterior or bileaflet prolapse presents a greater surgical challenge and is associated with higher rates of recurrent MR compared to isolated posterior leaflet prolapse. This increased technical difficulty is significant, as studies show that even mild residual regurgitation detected intraoperatively can more than quadruple the risk of long-term recurrence (18). Gamal et al. showed that MVR significantly reduces the recurrence rate of MR compared to MVP. However, no significant difference was found between the two approaches in terms of overall mortality or valve-related reoperation rates (19). Our findings confirm that MR is significantly more severe after MVP than MVR. However, there was no statistically significant difference in postoperative survival or valve-related reoperation rates between the two groups.

Beyond mitral regurgitation recurrence, anticoagulation-related hemorrhage represents the principal trade-off of prosthetic mitral valve replacement in this population. In this cohort, all major bleeding events (BARC grade ≥2) occurred in the MVR group (eight events pre-PSM; six events post-PSM; none in the MVP group), and mechanical prostheses, which require lifelong warfarin therapy, accounted for a substantial proportion of the MVR cohort (56/85 patients, 65.88%). Bleeding-related outcomes were concentrated within the MVR cohort. This discrepancy may largely stem from the repeated long-term anticoagulant administration required in patients with implanted mechanical heart valves. In contrast, major bleeding complications were absent in the patients who underwent MVP; the majority of these individuals only received anticoagulation therapy for a brief period following surgery. These observations suggest that for patients presenting with severe left-ventricular dilatation, clinicians should balance the bleeding risk associated with MVR (especially mechanical valve implantation) against the elevated likelihood of recurrent mitral regurgitation after valve repair. Individualized valve prosthesis choice, strict INR monitoring, and adequate patient counseling therefore remain critical for clinical decision-making.

Significant differences were observed in LVEF and GLS between the two groups. LVEF is a critical prognostic metric for assessing left ventricular systolic function and diagnosing heart failure, wherein reduced values often indicate myocardial injury (20). Recent evidence demonstrates the superior sensitivity of GLS over LVEF for the early detection of subclinical cardiac dysfunction and prediction of adverse outcomes (21, 22). GLS is a valuable tool for risk stratification, effectively predicting cardiovascular events and overall survival. It is particularly useful for detecting subclinical dysfunction when LVEF is preserved, and its reduction is strongly linked to adverse outcomes (23). Our findings show that, despite no significant difference in short-to-mid-term mortality, the numerically higher rate in the MVR cohort is notable. The significant differences in LVEF and GLS, both strongly linked to adverse outcomes, are notable. A reduced GLS, indicating early dysfunction prior to LVEF decline, suggests a potentially poorer long-term prognosis for patients who undergo MVR (24). Given the non-significant survival difference, the limited number of events at late follow-up, and the exploratory nature of these secondary echocardiographic endpoints, this interpretation should be regarded as hypothesis-generating and requires confirmation in prospective studies with protocolized serial strain assessment.

In summary, while MVP and MVR showed comparable postoperative mortality and valve-related reoperation rates, MVP was associated with a higher MR recurrence. The significant differences in left ventricular function (LVEF and GLS) suggest superior cardiac preservation with MVP and a potential long-term disadvantage with MVR, warranting further validation.

## Limitation

This single-center retrospective study has several limitations. First, the relatively modest sample size and lack of stratification according to important clinical and anatomical characteristics, such as age, prolapse location, and left ventricular size, may limit the generalizability of our findings. Second, baseline GLS and the specific location of leaflet prolapse (anterior, posterior, or bileaflet) were not incorporated into the propensity score matching model, leaving the possibility of residual confounding. Third, the limited sample size and number of events during long-term follow-up may have reduced the statistical power to detect differences in late survival, particularly in subgroup analyses. Fourth, follow-up echocardiographic parameters were obtained from the most recent available outpatient echocardiogram rather than at a predefined postoperative time point, and the interval between surgery and the last echocardiographic assessment was not separately recorded. Therefore, the observed between-group differences in LVEF and GLS reflect the most recent available cardiac functional status rather than a time-matched postoperative assessment and should be interpreted as hypothesis-generating. Larger prospective multicenter studies with protocolized and serial GLS assessments, standardized postoperative echocardiographic follow-up, and more comprehensive adjustment for clinical and anatomical characteristics are warranted to validate these findings.

## Conclusion

MVP was associated with a higher recurrence of MR, while MVR had a greater risk of postoperative hemorrhagic complications associated with oral anticoagulation. Although no significant differences were found in postoperative LVEDD, NYHA class, or mid-term overall survival rate, MVP may provide superior preservation of subclinical LV systolic function (LVEF and GLS). These findings suggest that MVP remains a favorable strategy when anatomically feasible, though long-term clinical trial validation is required.

## Data Availability

The original contributions presented in the study are included in the article/Supplementary Material, further inquiries can be directed to the corresponding authors.
